# Cardiovascular Effects of Calcium Supplements

**DOI:** 10.3390/nu5072522

**Published:** 2013-07-05

**Authors:** Ian R. Reid

**Affiliations:** Faculty of Medical and Health Sciences, University of Auckland, Auckland 1142, New Zealand; E-Mail: i.reid@auckland.ac.nz; Tel.: +64-9-3737-599 (ext. 86259); Fax: +64-9-308-2308

**Keywords:** calcium, myocardial infarction, stroke, osteoporosis

## Abstract

Calcium supplements reduce bone turnover and slow the rate of bone loss. However, few studies have demonstrated reduced fracture incidence with calcium supplements, and meta-analyses show only a 10% decrease in fractures, which is of borderline statistical and clinical significance. Trials in normal older women and in patients with renal impairment suggest that calcium supplements increase the risk of cardiovascular disease. To further assess their safety, we recently conducted a meta-analysis of trials of calcium supplements, and found a 27%–31% increase in risk of myocardial infarction, and a 12%–20% increase in risk of stroke. These findings are robust because they are based on pre-specified analyses of randomized, placebo-controlled trials and are consistent across the trials. Co-administration of vitamin D with calcium does not lessen these adverse effects. The increased cardiovascular risk with calcium supplements is consistent with epidemiological data relating higher circulating calcium concentrations to cardiovascular disease in normal populations. There are several possible pathophysiological mechanisms for these effects, including effects on vascular calcification, vascular cells, blood coagulation and calcium-sensing receptors. Thus, the non-skeletal risks of calcium supplements appear to outweigh any skeletal benefits, and are they appear to be unnecessary for the efficacy of other osteoporosis treatments.

## 1. Introduction

During the 1980s and 1990s, calcium supplements became widely used in the prevention and treatment of osteoporosis. Probably a number of factors contributed to their popularity, including evidence from calcium balance studies that increased intakes were associated with more positive balances, and the absence of other clearly effective therapies for managing osteoporosis. It is now clear that the positive findings from balance studies do not translate into either substantially increased bone density or reduced fracture risk in those who habitually take large amounts of calcium in their diet [1,2]. Indeed, the rationale for believing that large intakes of a single component of a connective tissue might contribute to an increased mass of that tissue can be questioned. However, there is now clear evidence that calcium supplements have small positive effects of bone mineral density in randomized, controlled trials [3], and they appear to reduce total fracture risk by as much as 10%, although they may actually *increase* hip fractures [4,5].

## 2. Clinical Trials & Meta-Analyses

While the bone effects of calcium supplementation have been clarified through a series of clinical trials, there has been ongoing interest in their effect on other organs, such as the cardiovascular system. Calcium supplements produce small beneficial effects on blood pressure [6] and on circulating cholesterol fractions [7] which might, over time, result in reduced cardiovascular events. On the basis of this hypothesis, we pre-specified cardiovascular events as secondary endpoints in a randomized, controlled trial of calcium supplements in normal postmenopausal women, and found, to our surprise, that the risk of myocardial infarction was significantly increased in the supplemented participants [8]. While such a finding could arise by chance, accumulating evidence of adverse effects of calcium supplements in patients with chronic kidney disease caused us to take these findings seriously and to undertake a meta-analysis of the effects of calcium supplementation on cardiovascular events across all the trials that have collected these data. To this end, we contacted the authors of all qualifying studies and obtained data in 11,921 randomized subjects, 93% of all subjects participating in trials of calcium monotherapy [9]. This analysis demonstrated a 27% increase in risk of myocardial infarction (*p* = 0.04) and non-significant upward trends in stroke (relative risk 1.12, *p* = 0.25) and death (relative risk 1.07, *p* = 0.26). Contemporaneously, Wang *et al.* undertook a similar meta-analysis, but only accessed published data [10]. They found a relative risk of cardiovascular events of 1.14 (95% CI 0.92–1.41) in a cohort of almost 4000 subjects, effectively a subset of our meta-analysis.

These findings provide confirmation of the adverse effects found in the Auckland Calcium Study, and showed a remarkable consistency across all the studies. However, the Women’s Health Initiative investigators had already published an assessment of the effects of calcium plus vitamin D on cardiovascular events, and had concluded that no significant effect was demonstrable [11]. The Women’s Health Initiative differed from the studies in our meta-analysis in that the population studied was about 10 years younger, the intervention included vitamin D as well as calcium, and more than half of the subjects entering the study were already taking their own calcium supplements (and continued to do so during trial in addition to the medication allocated as part of the study). Any one of these three differences could have accounted for the different findings in that study. Accordingly, we proposed an analysis which involved looking for an interaction between calcium supplement use at the time of randomization and the effect of the trial intervention on cardiovascular disease. The analysis plan was finalized and approved by the NHLBI, before the data were released to us, and the NHLBI approved the paper before its submission for publication. This analysis demonstrated that there were significant interactions, indicating that supplementation with calcium plus vitamin D significantly increased the risk of myocardial infarction in those who were calcium supplement naive but showed no such effect in those who were already taking calcium supplements (and just having an increase in supplement dose). Based on this finding, we concluded that co-administration of vitamin D with calcium did not abrogate the adverse effects on cardiovascular disease, but that the inclusion of a large number of women already taking calcium had obscured some of the effects of the trial intervention in the Women’s Health Initiative [12].

**Figure 1:**
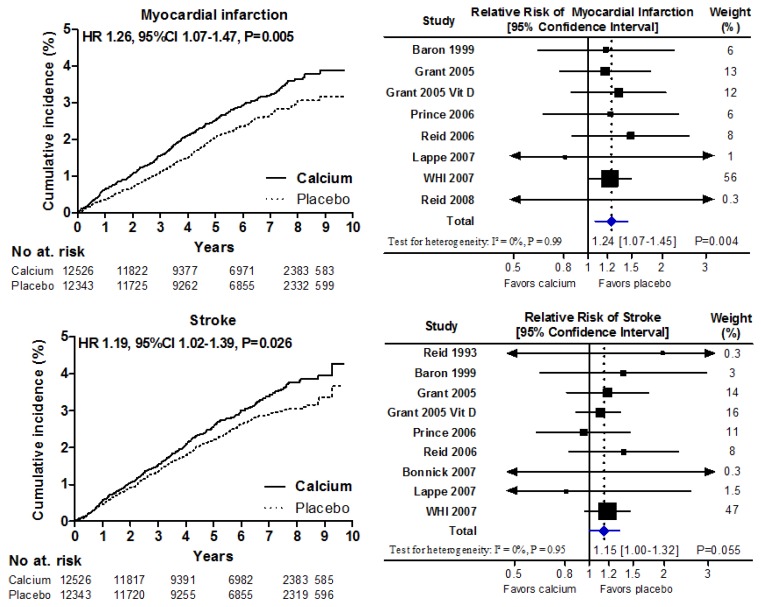
Patient-level meta-analyses of the effect of calcium supplements with or without vitamin D on cardiovascular events (left-hand panels) and corresponding trial-level meta-analyses (right-hand side). Patient-level data show the time-to-first event analyses for 24,869 participants in 5 trials of calcium supplements, and Women’s Health Initiative (WHI) calcium and vitamin D (CaD) participants not taking personal calcium supplements at baseline. Trial-level analyses show data for 28,072 participants in 8 trials of calcium supplements where complete trial-level data were available, together with data for WHI CaD participants not taking personal calcium supplements at baseline. One study randomized participants to calcium, CaD, or placebo [13]. For this analysis, we pooled the outcomes from both the calcium and CaD arms. Abbreviations: Grant 2005 is the RECORD study calcium *versus* placebo arms, and Grant 2005 Vit D is the RECORD study calcium plus vitamin D *versus* vitamin D plus placebo arms [14]. From Bolland *et al.* [12].

Following on from this analysis, we carried out a meta-analysis of all studies of either calcium or vitamin D for which cardiovascular event data are available (Figure 1). This demonstrated that those studies using calcium plus D have very similar effects on myocardial infarction risk to those using calcium alone, and that there is a 24%–26% increase in risk of myocardial infarction (*p* = 0.005) based on a trial population of more than 28,000 subjects. The increase in risk of stroke appears to be less (15%–19%) and sits at the borderline for statistical significance.

Sambrook *et al*. [15,16] have recently reported a study which randomized frail elderly subjects to control, daily sunlight exposure, or daily sunlight plus calcium supplements. Two hundred and eighteen deaths occurred during follow-up in the total patient population of 602. When comparing the calcium plus sunlight with the sunlight alone group, the hazard ratio for death was 1.47 (95% CI 1.05, 2.06) and that for cardiovascular death was 1.76 (1.10, 2.82). This suggests that in high risk populations, the increase in cardiovascular events does translate into increased mortality.

The results of these meta-analyses have led a number of investigators to assess the association between cardiovascular risk and calcium supplement use in observational cohorts. The Kuopio Osteoporosis Study of 10,555 older women followed for seven years, showed a hazard ratio for coronary heart disease in calcium supplement users of 1.24 (95% CI 1.02, 1.52) [17]. More recently, the EPIC-Heidelberg cohort of 23,980 adults followed for eleven years showed a hazard ratio for myocardial infarction of 1.86 (95% CI 1.17, 2.96) for calcium supplement use [18].

## 3. Mechanisms

While the trial data make a compelling case for the adverse cardiovascular effects of calcium supplements, the mechanism remains open to debate. *In vitro* cultures of vascular smooth muscle cells have been shown to exhibit calcification when the calcium concentration in the media is increased [19]. This is not a simple physicochemical process, since it involves increased numbers of matrix vesicles which form within the vascular smooth muscle cells and are subsequently extruded, resulting in extracellular deposition of calcium. Increased exposure of vascular smooth muscle cells to calcium results in altered gene expression and in the cells expressing a more osteoblastic phenotype. The cells of the arterial wall express calcium receptors, which may be involved in these changes. Increased vascular risk has been found in individuals with mutations of the calcium sensing receptor, which supports this possible pathogenic pathway [20].

**Figure 2:**
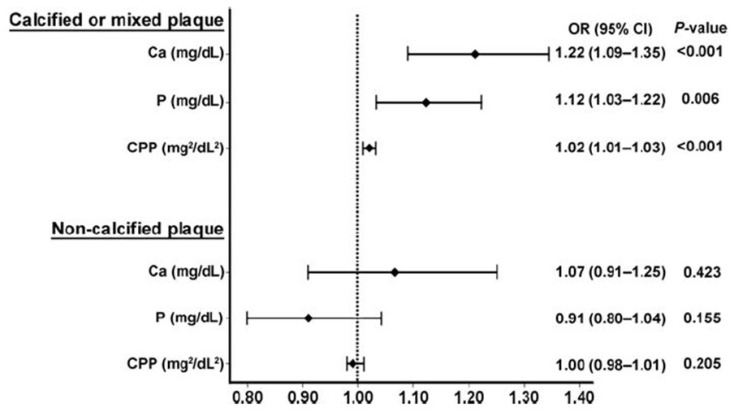
The association of serum calcium, phosphate, and calcium–phosphate product with the presence of coronary artery disease, divided into calcified or mixed plaque, and non-calcified plaque. Plaque was measured by cardiac computed tomography in 7553 Korean adults. From Shin *et al.* [23], used with permission.

There is now a substantial epidemiological literature which supports an association between circulating calcium levels and vascular risk. Studies in normal older populations have demonstrated that, as calcium levels increase within the normal range, there are increases in carotid artery plaque thickness [21], the likelihood of abdominal aortic calcification [22], the extent of coronary artery calcification [23], the incidence of cardiovascular events [24,25], and in total mortality [26,27]. Figure 2 demonstrates one such recent study of 7553 Korean adults, which assessed the association of serum calcium and phosphate levels on coronary artery atherosclerosis, as determined by computed tomography. The odds ratio of calcified or mixed plaque was 1.22 (*p* < 0.001) for each increase in serum calcium of 1 mg/mL [23].

The key factors linking these epidemiological data to the effects of calcium supplements are the abrupt increase in serum calcium which occurs following supplement ingestion. For example, Karp [28] has shown increases in ionized calcium of 0.07 mmol/L four hours after a 1 g calcium supplement, a change which is certainly large enough to increase coronary artery plaque and vascular event rates based on the observational data discussed above. In contrast, calcium-rich meals cause minimal perturbation of serum calcium levels, presumably because the calcium load is absorbed slowly, as a result of the presence of fat and protein in the meal, and because dietary calcium is usually taken in smaller boluses than the 500–1000 mg doses typical of supplement use. This suggests that calcium supplements produce non-physiological effects on serum calcium which adversely impact on vessel calcification. They may also cause subtle changes to other vascular mediators, such as the stability of atherosclerotic plaques, platelet function (via the calcium sensing receptor on platelets) and at a number of points within the coagulation cascade, in which calcium is a critical cofactor.

## 4. Conclusions

Thus, individuals considering taking or prescribing calcium supplements are left with a conundrum. There is equivocal evidence of benefit to bone health which needs to be balanced against steadily increasing evidence of adverse cardiovascular risk. This balance was assessed in the Bolland meta-analysis of calcium monotherapy, which calculated that treating 1000 people with calcium supplements for five years would cause an additional 14 myocardial infarctions, 10 strokes and 13 deaths, while preventing 26 fractures [9]. In younger subjects, the absolute numbers are smaller, but the balance remains negative [12]. Thus, the available data suggest that the widespread use of calcium supplements in older individuals is doing more harm than good and should be abandoned. The beneficial effects on bone density of calcium supplementation probably do not arise from treating a deficiency of dietary calcium, but rather the boluses act (via calcitonin and parathyroid hormone) as a weak antiresorptive therapy. Now that we have free access to much more potent antiresorptive therapies, it would seem sensible to target these to individuals at high fracture risk, and to encourage a balanced diet as the preferred way of ensuring that adequate calcium is available for normal mineralization of bone. The absence of a clinically significant association between calcium intake, bone density and fracture risk over the range seen in Western populations, suggests that calcium is not a critical determinant of bone health in most individuals taking a balanced Western diet.
